# Pre-cannulation ventilation duration and the trajectory of venovenous extracorporeal membrane oxygenation in COVID-19-related acute respiratory distress syndrome: a nationwide competing-risks cohort study

**DOI:** 10.1007/s10047-026-01600-8

**Published:** 2026-10-08

**Authors:** Takahiro Masuda, Akihiro Hirakawa

**Affiliations:** 1 Department of Anesthesiology, Institute of Science Tokyo Hospital, 1-5-45 Yushima, Bunkyo-ku, Tokyo, 113-8510 Japan; 2 Department of Clinical Biostatistics, Graduate School of Medical and Dental Sciences, Institute of Science Tokyo, 1-5-45 Yushima, Bunkyo-ku, Tokyo, 113-8510 Japan

**Keywords:** Extracorporeal membrane oxygenation, Mechanical ventilation, Decannulation, Competing risks, COVID-19

## Abstract

**Purpose:**

Pre-cannulation ventilation duration is often considered in selecting candidates for venovenous extracorporeal membrane oxygenation, but its relationship with the subsequent support trajectory is uncertain. We examined associations with first liberation, death before liberation, and time on support.

**Methods:**

We analyzed a nationwide Japanese registry of patients receiving venovenous extracorporeal membrane oxygenation for COVID-19-related acute respiratory distress syndrome. First liberation and death before liberation were treated as competing transitions over 90 days. Cause-specific Cox models were stratified by epidemic period and adjusted for age, sex, and body mass index. Expected time on support was calculated from state-occupancy probabilities.

**Results:**

Among 1,420 patients, the adjusted hazard ratio for first liberation relative to 0–1 days was 0.87 (95% confidence interval 0.72–1.05) for 2–3 days, 0.65 (0.54–0.78) for 4–7 days, 0.41 (0.32–0.52) for 8–14 days, and 0.58 (0.34–0.98) for at least 15 days. The association was strongest during the first 14 days. Death before liberation, which does not represent overall mortality because follow-up after liberation was short, showed no monotonic gradient below 15 days. Expected time on support was 26.9 days after more than 7 days of ventilation and 18.4 days after 7 days or less.

**Conclusions:**

Among selected recipients, longer pre-cannulation ventilation, particularly 8–14 days, was associated with slower early liberation and longer support. It may inform the anticipated course and resource requirements after cannulation, but does not define a candidacy threshold.

**Supplementary Information:**

The online version contains supplementary material available at https://doi.org/10.1007/s10047-026-01600-8.

## Introduction

Venovenous extracorporeal membrane oxygenation (VV-ECMO) provides temporary artificial lung support for selected patients with severe acute respiratory distress syndrome (ARDS). During the coronavirus disease 2019 (COVID-19) pandemic, its use expanded rapidly, and large international cohorts reported in-hospital mortality of approximately 40% [1–3]. Because VV-ECMO requires a circuit, specialized staff, and prolonged intensive care, decisions made at cannulation concern not only survival but also the likely duration and manner of leaving support, which bear on communication with families and on the organization of ECMO services [4].

The duration of invasive mechanical ventilation before cannulation is commonly considered in patient selection. It contributes to the RESP and PRESERVE risk scores [5, 6], and Extracorporeal Life Support Organization guidance lists more than 7 days of ventilation at high ventilator settings among relative contraindications, while acknowledging variation among centers [7]. Ventilation for more than 7 days was also an exclusion criterion in the EOLIA trial [8]. Evidence in COVID-19-related ARDS has nevertheless been inconsistent. Several cohorts found longer pre-ECMO ventilation to be associated with mortality [9, 10], whereas others questioned a fixed cutoff [11–14]. One multicenter study also linked additional ventilator days to longer ECMO support without a clear difference in survival [15].

Most previous studies summarized outcome as mortality or survival [16]. Those endpoints do not distinguish whether longer pre-cannulation ventilation is associated primarily with death during support, delayed liberation, or both. First liberation and death before liberation are mutually exclusive first transitions out of the ECMO state and can be examined within a competing-risks framework [17, 18]. We therefore used a nationwide Japanese registry to examine the association of pre-cannulation ventilation duration with each first transition and with the expected time spent on VV-ECMO.

## Methods

### Study design and data source

We conducted a retrospective cohort study using the Cross ICU Searchable Information System (CRISIS), managed by Japan ECMOnet for COVID-19. CRISIS is a multicenter prospective registry established during the pandemic to capture clinical data on severe COVID-19 across Japan and covers more than 70% of intensive care units in the country [9]. The ethics committee of Japan ECMOnet for COVID-19 approved this secondary analysis (approval number 2024-002) with opt-out consent, and the dataset was de-identified. Reporting follows the STROBE statement [19].

### Study population

We included patients aged 18 years or older with laboratory-confirmed COVID-19 who received VV-ECMO for ARDS, defined according to the Berlin criteria [20], between February 2020 and February 2023. Patients were excluded when the date of ECMO initiation was missing or follow-up from that date could not be determined, when pre-cannulation ventilation duration was missing or implausible (negative or longer than 120 days), or when the recorded outcome date preceded the recorded decannulation date (Supplementary Fig. S1). The time origin was ECMO initiation. Pre-cannulation ventilation duration was measured before this origin and treated as a baseline covariate on the ECMO time scale.

### Exposure

The exposure was the number of days from tracheal intubation to ECMO initiation. Because the distribution was discrete and concentrated near zero, the primary representation used categories of 0–1, 2–3, 4–7, 8–14, and at least 15 days, with 0–1 days as the reference. We also dichotomized the exposure at 7 days to correspond with existing risk scores and guidance. A 10-day dichotomy and a restricted cubic spline with prespecified knots at 1, 3, 7, and 14 days, truncated at 21 days, were used in sensitivity analyses (Supplementary Fig. S2).

### Outcomes

We considered two mutually exclusive first transitions from ECMO initiation: first liberation from ECMO and death before first liberation. Liberation was identified from the recorded date of decannulation. The registry recorded a single ECMO initiation date and a single decannulation date per patient; re-cannulation and subsequent ECMO runs were not captured. First liberation therefore denotes the first recorded decannulation and does not necessarily indicate sustained liberation from ECMO. For a patient recorded as having died, liberation was classified as occurring first when decannulation preceded the outcome date by more than 2 days; otherwise, the patient was classified as having died before liberation because same-day decannulation commonly reflects circuit removal at death. Sensitivity analyses used thresholds of more than 0, 2, and 7 days. Patients still receiving ECMO at the end of observation or lost to follow-up were censored. We estimated the cumulative incidence of each first transition, cause-specific hazards, and the expected time spent on ECMO within 90 days.

Survival after first liberation was not analyzed, because the registry recorded an outcome-ascertainment date rather than a fixed follow-up period: among patients liberated alive, median observation after decannulation was 7 days, only 5.3% were observed through day 90, and observation differed by exposure group. Deaths after first liberation were therefore not compared, and 90-day all-cause mortality could not be estimated reliably.

### Statistical analysis

Continuous variables are presented as medians with interquartile ranges and categorical variables as counts with percentages. Characteristics across exposure categories were compared using standardized mean differences. Death before first liberation and first liberation were analyzed as competing events [17, 18]. Cumulative incidence functions were estimated with the Aalen-Johansen estimator [21]. Cause-specific hazard ratios were estimated with Cox models that censored the competing transition, stratified by epidemic period and adjusted for age, sex, and body mass index. Epidemic period, defined as six intervals of ECMO initiation, was included as a stratification variable rather than as an exposure of interest, to account for changes over time in viral variants, vaccination, treatment, and thresholds for cannulation. Stratification rather than covariate adjustment was used because period did not satisfy the proportional-hazards assumption. Because patients treated in the same region may share centers, referral pathways, and practice patterns, their outcomes may be correlated. Robust standard errors clustered by prefecture were therefore used so that confidence intervals do not assume independence among such patients. Center identifiers were not available in the dataset.

The categorical exposure was assessed with a global Wald test on 4 degrees of freedom using the robust variance. No linear trend test was used because the categories were not equally spaced. Proportional hazards were assessed using Schoenfeld residuals. The assumption was not satisfied for the 7-day dichotomy in the model for first liberation; therefore, estimates were reported separately for days 0–14, 15–30, and 31–90 using episode splitting. Category-specific estimates were also reported for days 0–14. Analyses were performed with R version 4.5.2 [22].

### Expected time on ECMO

Expected time on ECMO within 90 days was calculated as the restricted mean time in the on-ECMO state by integrating the estimated probability of remaining on ECMO [23]. This quantity summarizes the expected duration of circuit occupancy per patient. Because time on ECMO ends with either first liberation or death before first liberation, a shorter expected time does not necessarily indicate faster recovery and should be interpreted together with the cumulative incidence of each transition. Category-specific values were unadjusted. For the 7-day dichotomy, we additionally obtained a covariate-standardized descriptive comparison using inverse-probability weighting [24]. Stabilized weights were estimated from age, sex, body mass index, and epidemic period, targeted the combined study population, and were truncated at the 99th percentile. Covariate balance was assessed with standardized mean differences [25] (Supplementary Table S1, Supplementary Fig. S3). Confidence intervals were obtained from 500 prefecture-cluster bootstrap replicates, with weights re-estimated within each replicate. These weighted estimates were used to assess robustness and were not given a causal interpretation.

### Missing data and sensitivity analysis

Pre-cannulation ventilation duration was missing or implausible in 30 patients, who were excluded without imputation of the exposure. Among analyzed patients, age was missing in 0.5%, sex in 0.4%, and body mass index in 2.5%; complete-case analysis was therefore used for the primary models. Multiple imputation by chained equations with 20 datasets served as a sensitivity analysis (Supplementary Table S2). The imputation model included analysis covariates, exposure, event indicators, and Nelson-Aalen estimates of the cumulative hazard for each competing transition [26]. Comorbidities, severity scores, respiratory mechanics, and vaccination status were not recorded. PaO2/FiO2 ratio and positive end-expiratory pressure (PEEP) at cannulation were missing in 14.2% and 13.9%, respectively, and were added only in a sensitivity analysis because they were measured after the exposure.

## Results

### Patients

During the study period, 1,453 patients received VV-ECMO for COVID-19-related ARDS. After exclusion of 33 patients with missing or implausible pre-cannulation ventilation duration or an outcome date preceding decannulation (Supplementary Fig. S1), the cohort comprised 1,420 patients. Median age was 58 years (interquartile range, 50–66), 1,117 patients (79.0%) were men, and median pre-cannulation ventilation duration was 1 day (interquartile range, 0–4), with 55.4% of patients at 0–1 days. The exposure categories comprised 787, 211, 275, 115, and 32 patients (Table 1); 147 patients (10.4%) had been ventilated for more than 7 days.

**Table 1:** Baseline characteristics by category of pre-ECMO ventilation duration

Characteristic	0–1 day (*n* = 787)	2–3 days (*n* = 211)	4–7 days (*n* = 275)	8–14 days (*n* = 115)	≥ 15 days (*n* = 32)	SMD	Missing *n* (%)
Age, years	57 [49–65]	58 [48–66]	59 [52–67]	59 [55–68]	62 [54–67]	0.23	7 (0.5)
Male sex	609 (77.9)	168 (79.6)	216 (78.5)	98 (85.2)	26 (83.9)	0.10	6 (0.4)
Body mass index, kg/m^2^	28.1 [24.7–31.9]	28.4 [24.6–32.0]	27.0 [24.3–30.4]	26.5 [23.9–29.0]	26.7 [24.5–28.5]	0.17	35 (2.5)
Pre-ECMO ventilation, days	0 [0–1]	2 [2–3]	5 [4–6]	10 [8–12]	17 [16–21]	—	0
PaO_2_/FiO_2_, mmHg	80 [68–100]	90 [70–106]	90 [72–110]	82 [69–100]	84 [69–124]	0.12	201 (14.2)
PEEP, cmH_2_O	12 [10–15]	12 [10–15]	12 [10–15]	12 [10–14]	12 [10–15]	0.14	198 (13.9)
Period of ECMO initiation						0.38	0
Jan–Sep 2020	112 (14.2)	53 (25.1)	70 (25.5)	26 (22.6)	6 (18.8)		
Oct 2020–Feb 2021	132 (16.8)	38 (18.0)	56 (20.4)	24 (20.9)	7 (21.9)		
Mar–Jun 2021	161 (20.5)	36 (17.1)	50 (18.2)	24 (20.9)	9 (28.1)		
Jul–Sep 2021	227 (28.8)	65 (30.8)	84 (30.5)	33 (28.7)	9 (28.1)		
Oct 2021–Jun 2022	65 (8.3)	14 (6.6)	10 (3.6)	7 (6.1)	0 (0)		
Jul 2022 onwards	90 (11.4)	5 (2.4)	5 (1.8)	1 (0.9)	1 (3.1)		

Data are median [interquartile range] for continuous variables and n (%) for categorical variables. Percentages are calculated among patients with non-missing data; the totals for sex do not equal the group sizes because sex was missing in 6 patients

*Abbreviations* *ARDS* acute respiratory distress syndrome, *ECMO* extracorporeal membrane oxygenation, *PEEP* positive end-expiratory pressure, *SMD* standardized mean difference

Characteristics at ECMO initiation are shown in Table 1 and, for the 7-day dichotomy, in Supplementary Table S3. Median age increased modestly across the categories, from 57 to 62 years. Measured hypoxemia at cannulation did not worsen monotonically with ventilation duration; median PaO2/FiO2 ratio was lowest in the 0-1-day category. Cannulation after prolonged ventilation became less frequent later in the pandemic. The proportion of patients ventilated for more than 7 days before cannulation was 12.0% (32 of 267) in the first period and 10.0% (42 of 418) in the period from July to September 2021, but fell to 7.3% (7 of 96) and 2.0% (2 of 102) in the two most recent periods (Supplementary Table S3).

### Transitions out of ECMO

**Fig. 1:**
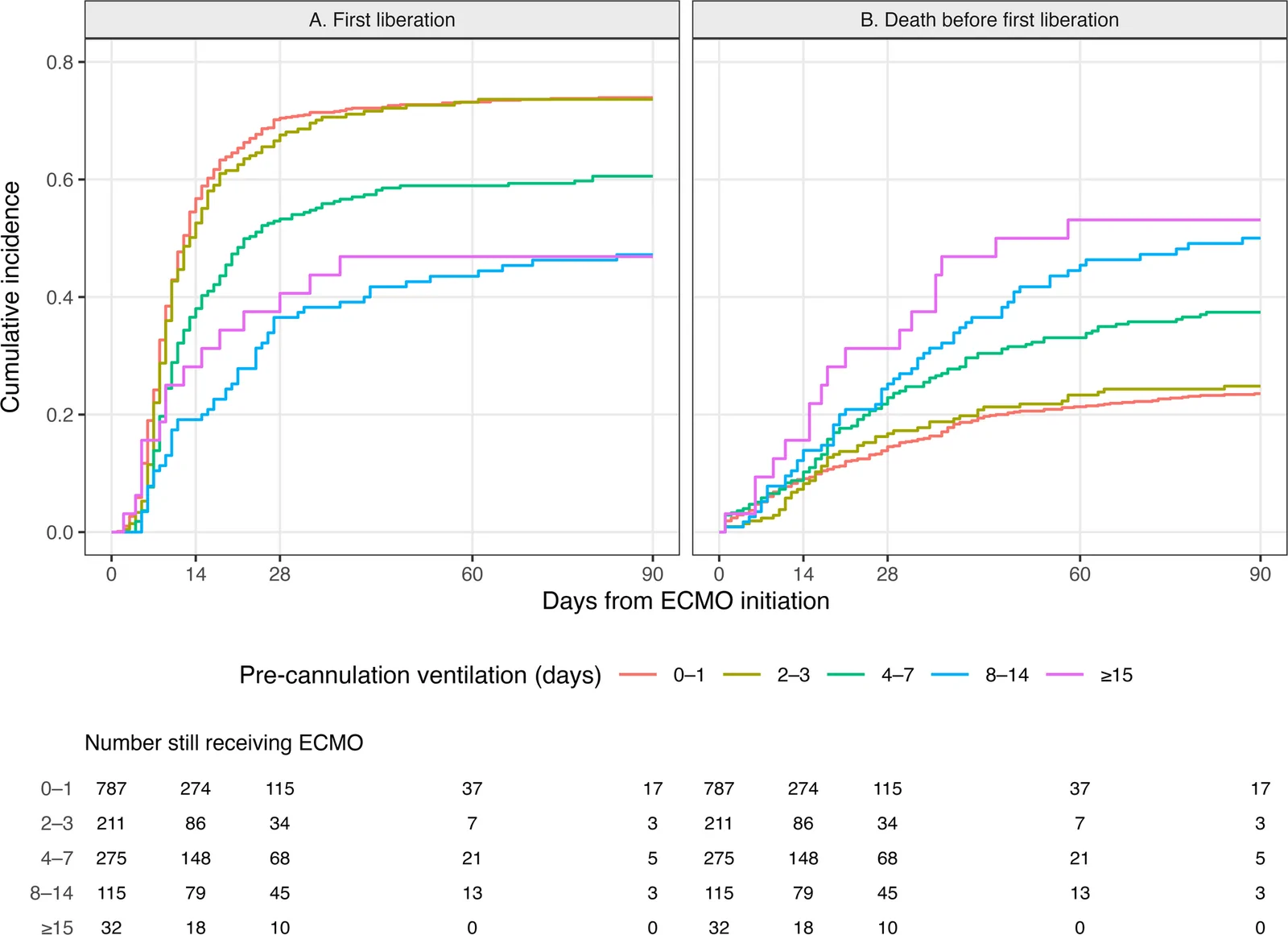
Cumulative incidence of first liberation (Panel **A**) and of death before first liberation (Panel **B**), by category of pre-ECMO ventilation duration. Aalen-Johansen estimates over 90 days from ECMO initiation, treating the two transitions as competing events. Numbers still receiving ECMO at 0, 14, 28, 60, and 90 days are shown beneath the panels. ECMO, extracorporeal membrane oxygenation

Within 90 days after ECMO initiation, 943 patients experienced first liberation, 396 died before first liberation, and 81 remained on ECMO at the end of observation or were lost to follow-up. The cumulative incidence functions are shown in Fig. 1 and, for the 7-day dichotomy, in Supplementary Fig. S4. At 90 days, cumulative incidence of first liberation declined from 0.74 in the 0-1-day category to 0.47 in the 8-14-day and at least 15-day categories, whereas that of death before first liberation rose from 0.24 to 0.53 (Table 3).

#### Rate of first liberation

Relative to 0–1 days, the adjusted cause-specific hazard ratio for first liberation over 90 days was 0.87 (95% confidence interval [CI], 0.72–1.05) for 2–3 days, 0.65 (95% CI, 0.54–0.78) for 4–7 days, 0.41 (95% CI, 0.32–0.52) for 8–14 days, and 0.58 (95% CI, 0.34–0.98) for at least 15 days (Table 2, Supplementary Fig. S5). The exposure categories were jointly associated with the liberation rate (chi-square 73.3 on 4 degrees of freedom; *P* < 0.001).

**Table 2:** Association between pre-ECMO ventilation duration and the two first transitions out of ECMO

Pre-ECMO ventilation	At risk at start of period	Events in period	Cause-specific hazard ratio (95% CI)
First liberation from ECMO, days 0–90			
0–1 day	787	559	1 (reference)
2–3 days	211	151	0.87 (0.72 to 1.05)
4–7 days	275	164	0.65 (0.54 to 0.78)
8–14 days	115	54	0.41 (0.32 to 0.52)
≥ 15 days	32	15	0.58 (0.34 to 0.98)
First liberation from ECMO, days 0–14			
0–1 day	787	434	1 (reference)
2–3 days	211	109	0.84 (0.65 to 1.09)
4–7 days	275	104	0.59 (0.46 to 0.76)
8–14 days	115	22	0.26 (0.17 to 0.40)
≥ 15 days	32	9	0.56 (0.34 to 0.91)
Death before first liberation, days 0–90			
0–1 day	787	172	1 (reference)
2–3 days	211	50	1.31 (1.04 to 1.65)
4–7 days	275	100	1.21 (0.97 to 1.51)
8–14 days	115	57	1.11 (0.81 to 1.53)
≥ 15 days	32	17	2.51 (1.43 to 4.41)
Seven-day dichotomy (> 7 versus ≤ 7 days)			
First liberation, days 0–90	1273 vs. 147	874 vs. 69	0.49 (0.39 to 0.63)
First liberation, days 0–14	1273 vs. 147	647 vs. 31	0.36 (0.24 to 0.53)
First liberation, days 15–30	474 vs. 95	177 vs. 24	0.63 (0.38 to 1.04)
First liberation, days 31–90	189 vs. 50	50 vs. 14	1.16 (0.73 to 1.85)
Death before first liberation, days 0–90	1273 vs. 147	322 vs. 74	1.15 (0.89 to 1.50)

Estimates are cause-specific hazard ratios from Cox models stratified by period of ECMO initiation and adjusted for age, sex, and body mass index, with robust standard errors accounting for clustering within prefectures

*Abbreviations* *CI* confidence interval, *ECMO* extracorporeal membrane oxygenation

The association was strongest early after cannulation. During days 0–14, the hazard ratio for the 8-14-day category was 0.26 (95% CI, 0.17–0.40) (Table 2). With the 7-day dichotomy, the hazard ratio for first liberation was 0.36 (95% CI, 0.24–0.53) during days 0–14, 0.63 (95% CI, 0.38–1.04) during days 15–30, and 1.16 (95% CI, 0.73–1.85) during days 31–90; the time-averaged estimate was 0.49 (95% CI, 0.39–0.63).

#### Death before first liberation

The exposure categories were also jointly associated with the rate of death before first liberation (chi-square 19.8 on 4 degrees of freedom; *P* < 0.001), but the category-specific estimates did not show a monotonic gradient below 15 days. Relative to 0–1 days, the hazard ratio ranged from 1.11 (95% CI, 0.81–1.53) to 1.31 (95% CI, 1.04–1.65) across the 2–3, 4–7, and 8-14-day categories, and was 2.51 (95% CI, 1.43–4.41) in the 32 patients ventilated for at least 15 days (Table 2). With the 7-day dichotomy, the hazard ratio was 1.15 (95% CI, 0.89–1.50).

#### Time on ECMO

Expected time on ECMO within 90 days rose from 17.4 days (95% CI, 16.0-18.8) after 0–1 days of ventilation to 29.1 days (95% CI, 25.0-33.2) after 8–14 days, and was 19.0 days (95% CI, 14.1–23.9) after at least 15 days (Table 3). The shorter time in the last category reflected exit from ECMO by either liberation or death and should not be interpreted as faster recovery.

**Table 3:** Ninety-day state occupancy and expected time on extracorporeal membrane oxygenation

Pre-cannulation ventilation	Cumulative incidence of first liberation at day 90	Cumulative incidence of death before first liberation at day 90	Expected time on ECMO within 90 days, days (95% CI)
By category of pre-cannulation ventilation			
0–1 day	0.74	0.24	17.4 (16.0–18.8)
2–3 days	0.74	0.25	17.6 (15.3–19.9)
4–7 days	0.61	0.37	21.9 (19.5–24.3)
8–14 days	0.47	0.50	29.1 (25.0–33.2)
≥15 days	0.47	0.53	19.0 (14.1–23.9)
By seven-day dichotomy			
≤7 days (*n* = 1,273)	0.71	0.27	18.4 (16.9–19.8)
>7 days (*n* = 147)	0.47	0.51	26.9 (23.7–31.3)
Difference, > 7 versus ≤ 7 days			8.5 (4.7–12.8)
≤7 days, inverse-probability weighted			18.2 (16.9–19.6)
>7 days, inverse-probability weighted			26.7 (23.6–31.2)
Difference, inverse-probability weighted			8.5 (5.0–13.0)

Expected time on ECMO is the unadjusted restricted mean time in the on-ECMO state over 90 days. Time on ECMO ends with either first liberation or death before first liberation; a shorter value therefore does not necessarily indicate faster recovery. Weighted estimates were standardized to the combined study population by inverse-probability weighting on age, sex, body mass index, and epidemic period, with 95% confidence intervals from 500 prefecture-cluster bootstrap replicates

*CI* confidence interval, *ECMO* extracorporeal membrane oxygenation

With the 7-day dichotomy, unadjusted expected time on ECMO was 18.4 days (95% CI, 16.9–19.8) after 7 days or less and 26.9 days (95% CI, 23.7–31.3) after more than 7 days, a difference of 8.5 days (95% CI, 4.7–12.8) (Table 3). Weighted estimates were nearly identical (difference, 8.5 days; 95% CI, 5.0–13.0). The proportion still receiving ECMO after day 30 was 14.8% (189/1,273) and 34.0% (50/147), respectively (Supplementary Fig. S6).

#### Sensitivity analyses

Adding PaO2/FiO2 ratio and PEEP at cannulation yielded hazard ratios of 0.53 (95% CI, 0.40–0.70) for first liberation and 1.22 (95% CI, 0.93–1.60) for death before liberation with the 7-day dichotomy. Hazard ratios for first liberation were 0.50 (95% CI, 0.39–0.64) and 0.47 (95% CI, 0.36–0.61) with classification thresholds of more than 0 and more than 7 days, and 0.53 (95% CI, 0.40–0.69) with a 10-day dichotomy. Multiple-imputation estimates were consistent with the complete-case analysis (Supplementary Table S2).

## Discussion

In this nationwide cohort of patients selected to receive VV-ECMO for COVID-19-related ARDS, longer pre-cannulation ventilation was associated most consistently with slower first liberation and longer circuit dependence, with the lowest liberation rate and the longest expected time on ECMO (29.1 days) in the 8-14-day category. The association was concentrated in the first 2 weeks after cannulation. In contrast, death before first liberation showed no monotonic gradient below 15 days, although the small group ventilated for at least 15 days had a higher hazard of death.

These findings add a transition-specific interpretation to earlier mortality-based studies. A previous analysis of this registry identified pre-ECMO ventilation duration as an independent prognostic factor in a single-endpoint model [9]. Other studies have reported either an association with mortality [10] or no clear survival disadvantage from delayed cannulation [11–14]. A multicenter report linked each additional ventilator day to prolonged ECMO support without a difference in survival [15]. By separating first liberation from death before liberation, the present analysis shows that the most coherent gradient was in the timing of liberation, a distinction that a binary survival endpoint cannot make.

The observed association should not be interpreted as the causal effect of waiting to initiate ECMO, and this limitation is central to the interpretation of the study. Pre-cannulation ventilation duration is an indirect marker that may reflect progression of lung injury, delayed referral, center-level practice, changing thresholds for cannulation, and selection of patients thought likely to remain eligible despite prolonged ventilation. The primary models adjusted only for age, sex, body mass index, and epidemic period, because respiratory mechanics, ventilator intensity, vasopressor requirement, organ dysfunction, coagulopathy, comorbidities, and the clinical reasons for delayed cannulation were not recorded in the registry. Measured hypoxemia at cannulation did not worsen monotonically with duration, and adjustment for PaO2/FiO2 ratio and PEEP did not materially alter the estimates, which argues against an explanation by gas-exchange severity alone. Nevertheless, residual confounding by unmeasured severity remains likely, and the estimates describe the course observed after cannulation rather than the effect of ventilation duration itself. Prolonged injurious ventilation is biologically compatible with slower recovery [27], but this registry cannot establish that mechanism.

The results are more informative for planning the course after cannulation than for deciding candidacy. Patients ventilated for more than 7 days spent approximately 8.5 additional expected days on ECMO, and one third were still receiving support at day 30. These differences translate into longer use of circuits, intensive care beds, and specialized personnel. Such considerations were especially visible during pandemic capacity constraints [28, 29], but anticipating circuit occupancy and staffing needs remains inherent to ECMO program management. The estimates may also help clinicians frame discussions with families about the likely pace of separation from support.

Two features of the pattern require caution. First, the group ventilated for at least 15 days contained only 32 patients and 15 first liberations, and its estimates should be interpreted with particular caution. Its shorter expected time on ECMO than the 8-14-day group did not indicate faster recovery, because time on ECMO ended with either liberation or death and this group left ECMO earlier through both transitions. Second, cause-specific hazards are conditional on remaining on ECMO. As deaths accumulate, the patients still eligible to experience liberation become an increasingly selected subset. This dynamic may contribute to the convergence of liberation hazards after day 30 and to the non-monotonic estimate in the longest-duration category.

The study does not identify a threshold beyond which VV-ECMO should be withheld, and the findings should not be read as showing that patients ventilated for longer periods were unsuitable candidates, for three reasons. First, the cohort is conditioned on having received support: patients considered for ECMO after prolonged ventilation but not cannulated are, by design, not represented, and those cannulated after 8 days or more were probably a highly selected subgroup, so the data cannot estimate how outcomes would have differed had cannulation been withheld or performed earlier. Second, a lower rate of liberation is not futility: 47% of patients in the 8-14-day category were liberated within 90 days, and death before liberation did not increase monotonically below 15 days. Third, the relevant ELSO criterion combines duration with high ventilator settings, and plateau pressure was not recorded, so that criterion itself could not be evaluated [7]. Among patients who were cannulated, approximately 1–2 weeks of prior ventilation marked a course of slower early liberation and longer circuit dependence.

This study has additional limitations. Follow-up after first liberation was short and differed between groups, preventing a reliable comparison of post-liberation deaths or 90-day all-cause mortality; death before first liberation is therefore distinct from overall mortality. Re-cannulation was not recorded, so first liberation does not imply sustained liberation. Classification of events close to decannulation required an operational rule, although results were stable across alternative thresholds. Epidemic period was a stratification variable rather than a comparison of interest; nevertheless, the distribution of pre-cannulation ventilation duration shifted substantially across periods, and only 2 patients ventilated for more than 7 days were treated in the most recent period, leaving residual imbalance after weighting and limiting the applicability of the estimates to later phases of the pandemic. Clustering was accounted for at the prefecture level because center identifiers were unavailable, so center-level variation in practice may not be fully captured. Finally, the extreme exposure categories were small, and the analysis was not designed to detect an optimal cutoff.

## Conclusions

Among patients selected to receive VV-ECMO for COVID-19-related ARDS, longer pre-cannulation ventilation was associated predominantly with slower early liberation and longer dependence on ECMO, with the lowest early liberation rate and the longest expected time on support after 8–14 days of ventilation. Because the cohort is conditioned on having received ECMO and important determinants of both cannulation timing and outcome were unmeasured, these associations describe the anticipated course after cannulation rather than a causal effect of ventilation duration. They may inform communication and resource planning, but they do not define an upper duration threshold for ECMO candidacy.

## Supplementary Information

Below is the link to the electronic supplementary material.

Supplementary Material 1: Supplementary Table S1. Covariate balance before and after inverse-probability weighting, for the seven-day dichotomy. Supplementary Table S2. Cause-specific hazard ratios from the complete-case analysis and after multiple imputation. Supplementary Table S3. Baseline characteristics by the seven-day dichotomy. Supplementary Fig. S1. Study flow diagram. Supplementary Fig. S2. Restricted cubic spline for first liberation, with knots specified a priori at 1, 3, 7, and 14 days. Supplementary Fig. S3. Distribution of stabilized inverse-probability weights. Supplementary Fig. S4. Cumulative incidence functions for the seven-day dichotomy. Supplementary Fig. S5. Cause-specific hazard ratios for first liberation, by category of pre-cannulation ventilation duration. Supplementary Fig. S6. Probability of remaining on ECMO, by the seven-day dichotomy.

## Data Availability

The data were used under license and may be available on reasonable request with permission from the CRISIS Management Committee.
