# Supplementary material for: Pre-cannulation ventilation duration and the trajectory of venovenous extracorporeal membrane oxygenation in COVID-19-related acute respiratory distress syndrome: a nationwide competing-risks cohort study
Source: J Artif Organs. 2026 Oct 8;29(4):67. doi: 10.1007/s10047-026-01600-8 (PMC13649870; doi:10.1007/s10047-026-01600-8)
Supplement: Supplementary file 1 [file 10047_2026_1600_MOESM1_ESM.pdf]

Supplementary material

Pre-cannulation ventilation duration and the trajectory of venovenous extracorporeal membrane oxygenation in COVID-19-related acute respiratory distress syndrome: a nationwide competing-risks cohort study

Supplementary Table S1. Covariate balance before and after inverse-probability weighting, for the seven-day dichotomy

Variable	SMD before weighting	SMD after weighting
Age	0.328	0.107
Male sex	0.161	0.076
Body mass index	−0.219	−0.030
Period: Jan–Sep 2020	0.088	0.041
Period: Oct 2020–Feb 2021	0.083	0.048
Period: Mar–Jun 2021	0.074	0.038
Period: Jul–Sep 2021	−0.020	0.011
Period: Oct 2021–Jun 2022	−0.093	−0.066
Period: Jul 2022 onwards	−0.296	−0.223

Weights were derived from a logistic model of ventilation for more than seven days on age, sex, body mass index and period of ECMO initiation. Stabilized weights targeting the average treatment effect were used and truncated at the 99th percentile.

Supplementary Table S2. Cause-specific hazard ratios from the complete-case analysis and after multiple imputation

Pre-cannulation ventilation	Complete case (n = 1,377)	Multiple imputation (n = 1,420)
**First liberation from ECMO, days 0–90**		
0–1 day	1 (reference)	1 (reference)
2–3 days	0.87 (0.72 to 1.05)	0.87 (0.72 to 1.05)
4–7 days	0.65 (0.54 to 0.78)	0.66 (0.55 to 0.79)
8–14 days	0.41 (0.32 to 0.52)	0.42 (0.31 to 0.55)
≥15 days	0.58 (0.34 to 0.98)	0.56 (0.33 to 0.93)
**First liberation from ECMO, days 0–14**		
0–1 day	1 (reference)	1 (reference)
2–3 days	0.84 (0.65 to 1.09)	0.83 (0.67 to 1.03)
4–7 days	0.59 (0.46 to 0.76)	0.60 (0.48 to 0.74)
8–14 days	0.26 (0.17 to 0.40)	0.27 (0.17 to 0.41)
≥15 days	0.56 (0.34 to 0.91)	0.47 (0.24 to 0.91)
**Death before first liberation, days 0–90**		
0–1 day	1 (reference)	1 (reference)
2–3 days	1.31 (1.04 to 1.65)	1.27 (0.92 to 1.76)
4–7 days	1.21 (0.97 to 1.51)	1.27 (0.98 to 1.64)
8–14 days	1.11 (0.81 to 1.53)	1.14 (0.83 to 1.56)
≥15 days	2.51 (1.43 to 4.41)	2.38 (1.43 to 3.98)
**Seven-day dichotomy (>7 versus ≤7 days)**		
First liberation	0.49 (0.39 to 0.63)	0.50 (0.39 to 0.64)
Death before first liberation	1.15 (0.89 to 1.50)	1.17 (0.90 to 1.51)

Estimates are cause-specific hazard ratios from Cox models stratified by period of ECMO initiation and adjusted for age, sex and body mass index, with robust standard errors accounting for clustering within prefectures.

Supplementary Table S3. Baseline characteristics by the seven-day dichotomy

**Characteristic**	**≤7 days (n = 1,273)**	**>7 days (n = 147)**	**SMD**	**Missing n (%)**
Age, years	57 [49–66]	60 [55–68]	0.31	7 (0.5)
Male sex	993 (78.3)	124 (84.9)	0.17	6 (0.4)
Body mass index, kg/m^2	27.9 [24.6–31.5]	26.5 [24.0–29.0]	0.22	35 (2.5)
Pre-cannulation ventilation, days	1 [0–3]	11 [9–14]	—	0
PaO_2/FiO_2 at cannulation, mmHg	84 [70–103]	83 [69–103]	<0.01	201 (14.2)
PEEP at cannulation, cmH_2O	12 [10–15]	12 [10–14]	0.11	198 (13.9)
Period of ECMO initiation			0.35	0
Jan–Sep 2020	235 (18.5)	32 (21.8)		
Oct 2020–Feb 2021	226 (17.8)	31 (21.1)		
Mar–Jun 2021	247 (19.4)	33 (22.4)		
Jul–Sep 2021	376 (29.5)	42 (28.6)		
Oct 2021–Jun 2022	89 (7.0)	7 (4.8)		
Jul 2022 onwards	100 (7.9)	2 (1.4)		

Data are median [interquartile range] for continuous variables and n (%) for categorical variables. Percentages are calculated among patients with non-missing data.

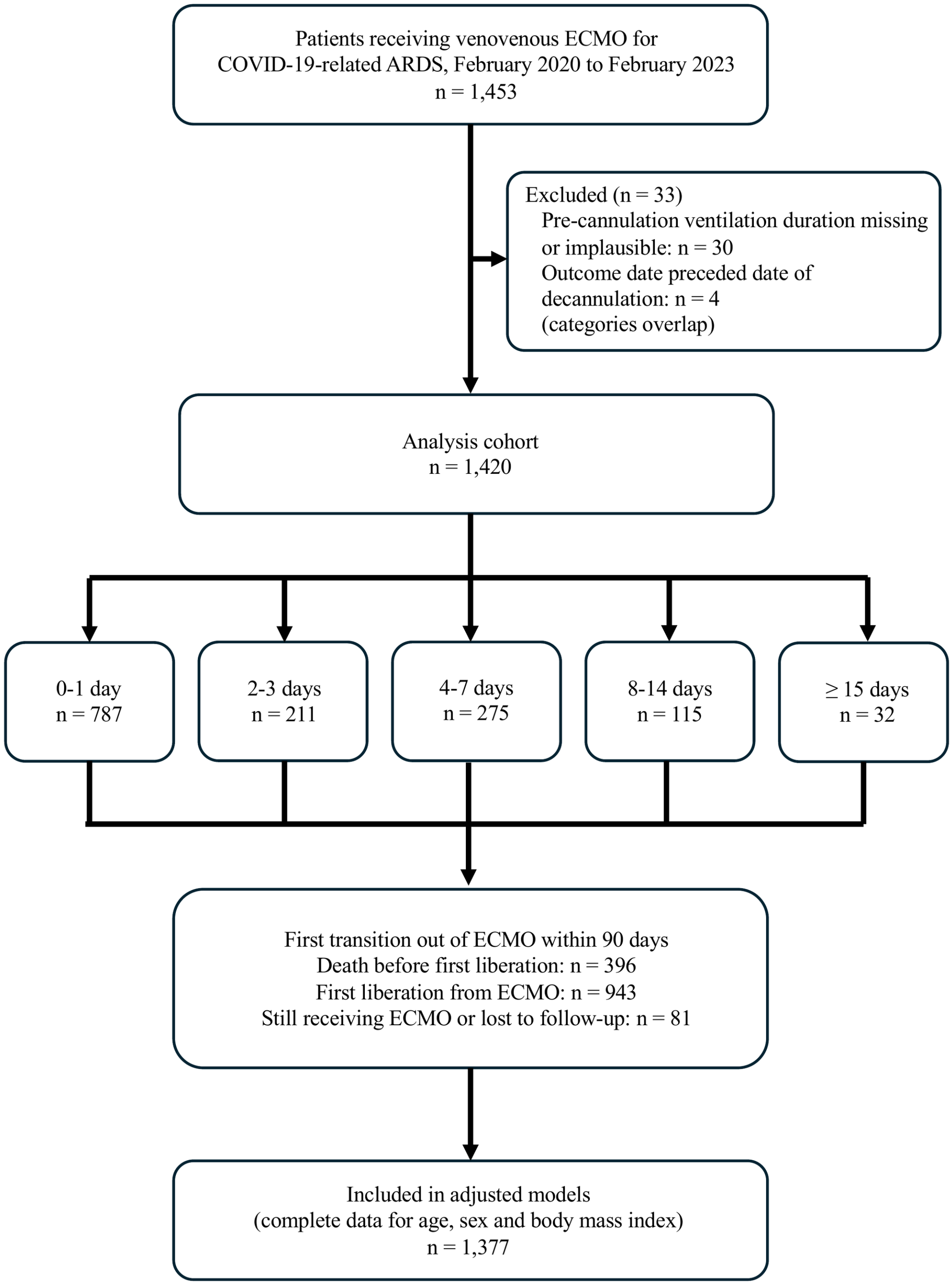

Supplementary Fig. S1. Study flow diagram. Numbers of patients at each stage, from the registry extract to the analysis cohort and the adjusted models. Exclusion categories overlap. ECMO, extracorporeal membrane oxygenation.

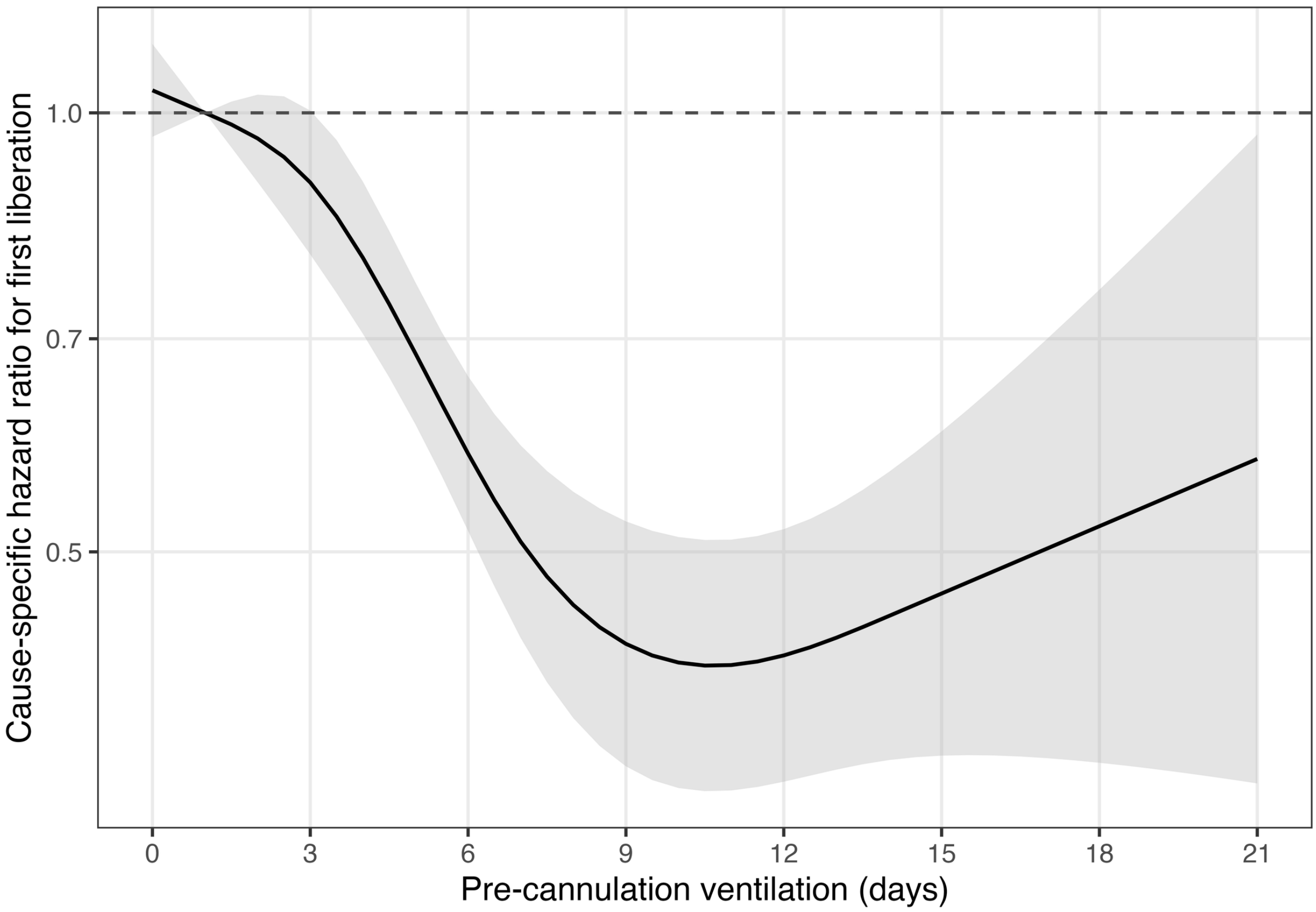

Supplementary Fig. S2. Restricted cubic spline for the cause-specific hazard of first liberation, with knots specified a priori at 1, 3, 7 and 14 days and one day as the reference. The curve is truncated at 21 days. The shaded band is the 95% confidence interval and the vertical axis is on a logarithmic scale. Estimates are from a Cox model stratified by period of ECMO initiation and adjusted for age, sex and body mass index. ECMO, extracorporeal membrane oxygenation.

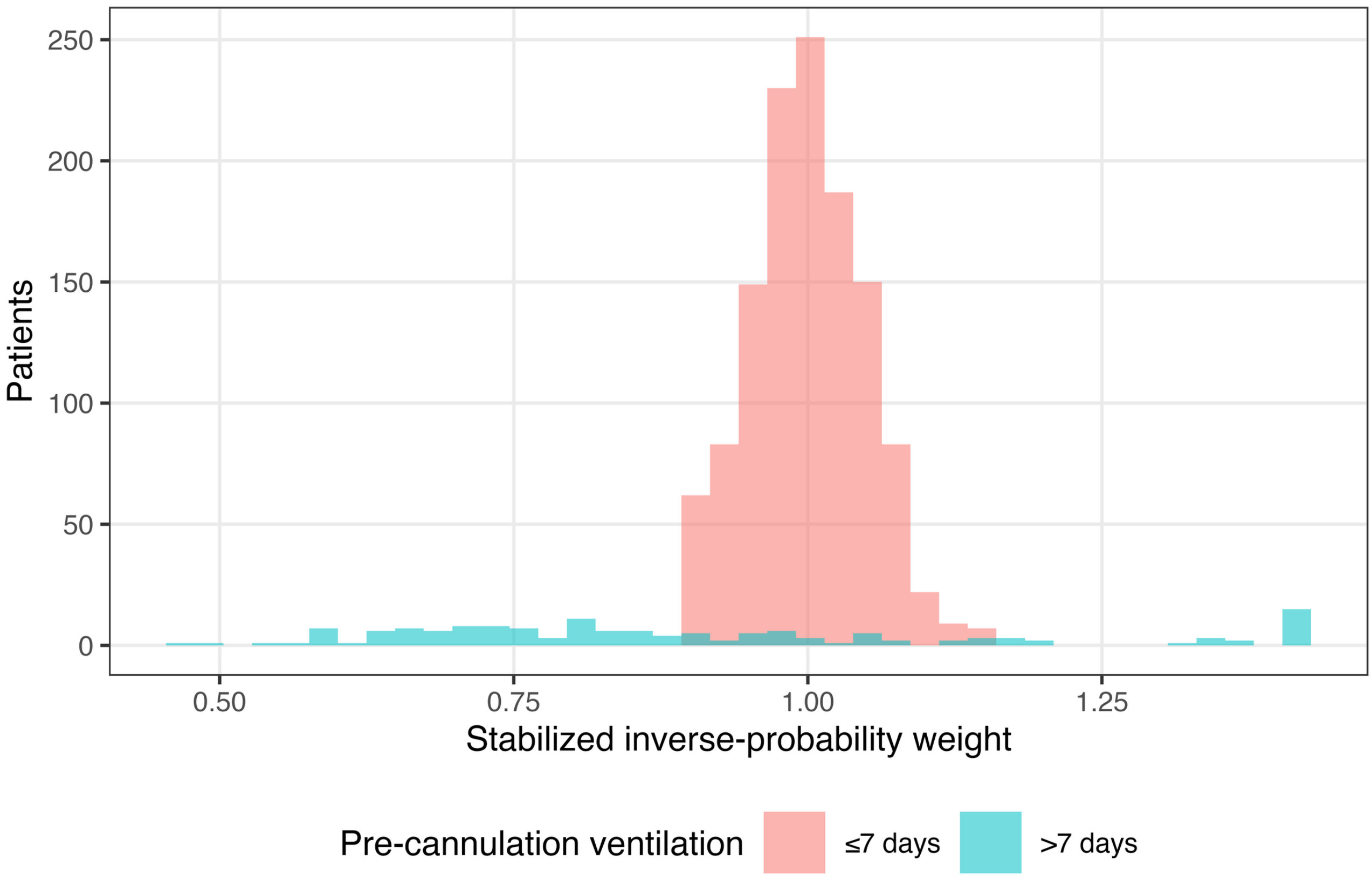

Supplementary Fig. S3. Distribution of stabilized inverse-probability weights, by the seven-day dichotomy. Weights were derived from a logistic model of ventilation for more than seven days on age, sex, body mass index and period of ECMO initiation, targeted the average treatment effect, and were truncated at the 99th percentile. ECMO, extracorporeal membrane oxygenation.

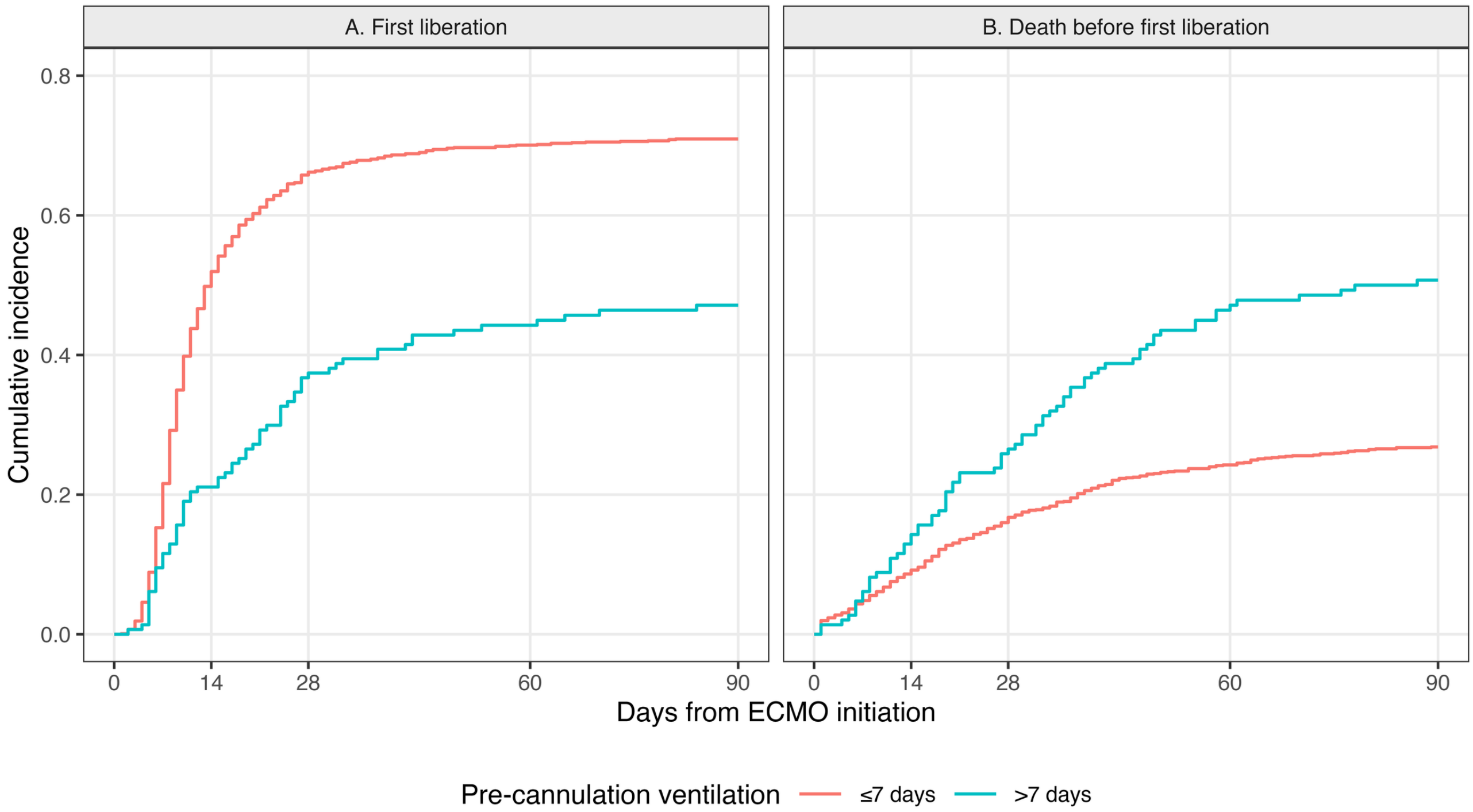

Supplementary Fig. S4. Cumulative incidence of first liberation and of death before first liberation, by the seven-day dichotomy. Aalen-Johansen estimates over 90 days from ECMO initiation, treating first liberation from ECMO (Panel A) and death before first liberation (Panel B) as competing events. ECMO, extracorporeal membrane oxygenation.

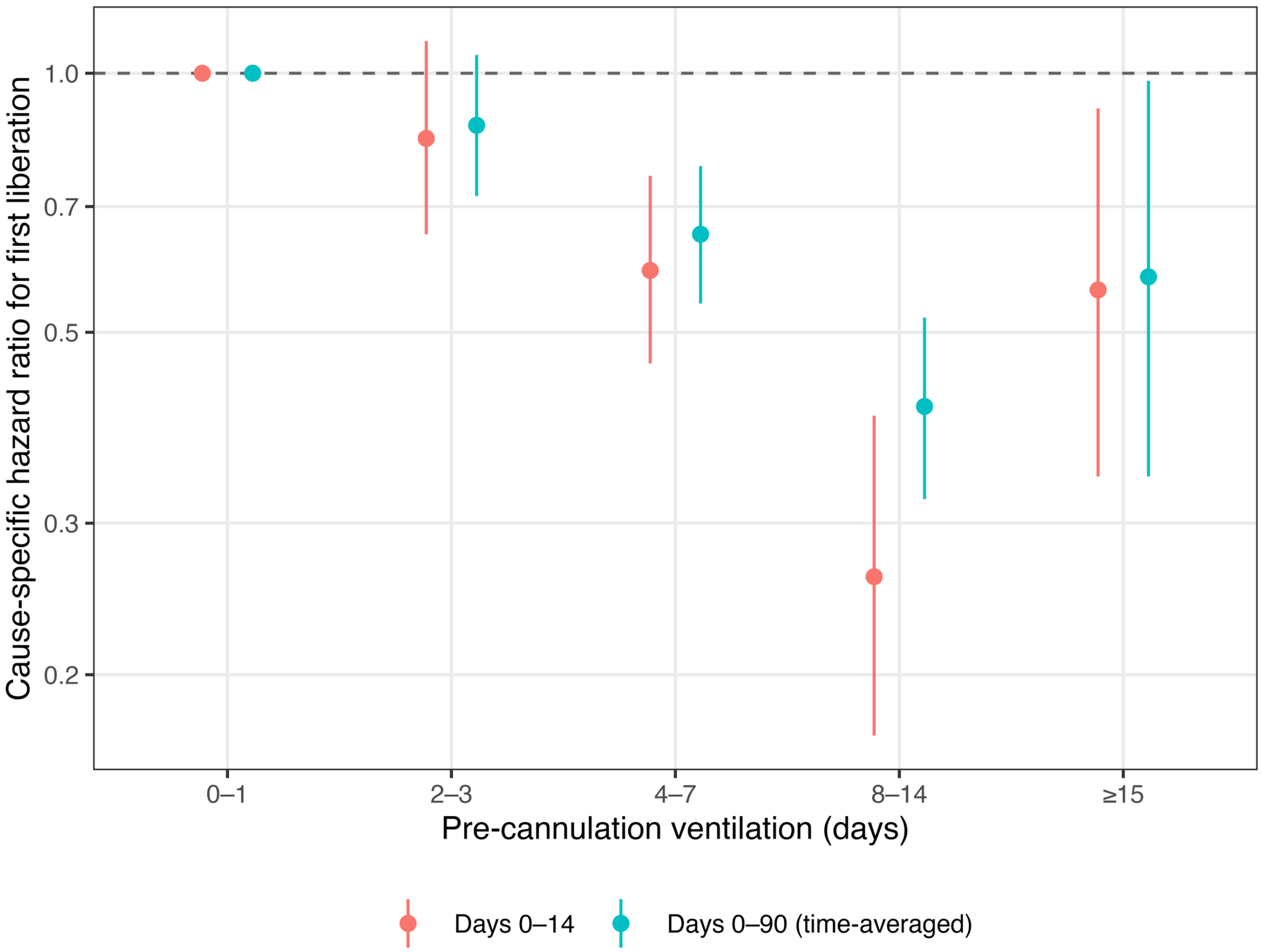

Supplementary Fig. S5. Cause-specific hazard ratios for first liberation, by category of pre-cannulation ventilation duration. Estimates are relative to 0–1 days, shown time-averaged over 90 days and within days 0–14. Vertical bars are 95% confidence intervals and the vertical axis is on a logarithmic scale. Estimates are from Cox models stratified by period of ECMO initiation and adjusted for age, sex and body mass index. ECMO, extracorporeal membrane oxygenation.

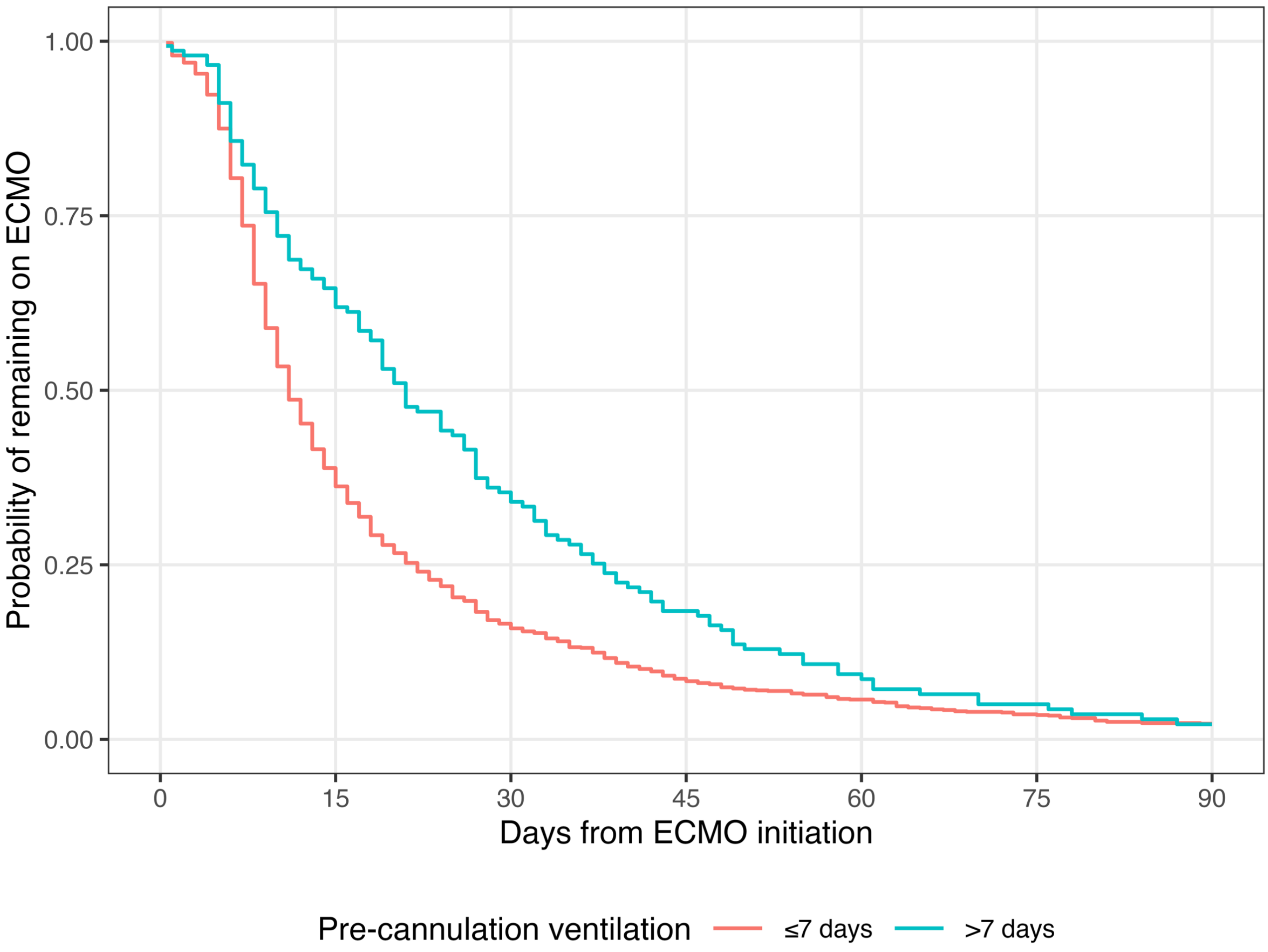

Supplementary Fig. S6. Probability of remaining on ECMO, by the seven-day dichotomy. Estimates over 90 days from ECMO initiation. Both ways of leaving ECMO were observed, so this probability is estimable without assumptions about survival after liberation. The area under each curve is the unadjusted expected time on ECMO within 90 days, 18.4 days for ventilation of seven days or less and 26.9 days for longer ventilation. Because time on ECMO ends with either first liberation or death before first liberation, a longer time on ECMO reflects slower exit through both transitions and should not be read as a measure of recovery alone. ECMO, extracorporeal membrane oxygenation.
